# Impact of Auxins on Vegetative Propagation through Stem Cuttings of* Couroupita guianensis* Aubl.: A Conservation Approach

**DOI:** 10.1155/2016/6587571

**Published:** 2016-12-19

**Authors:** Mahipal S. Shekhawat, M. Manokari

**Affiliations:** ^1^Department of Plant Science, M.G.G.A.C., Mahe, Pondicherry, India; ^2^Department of Botany, K.M. Centre for Postgraduate Studies, Pondicherry, India

## Abstract

The present study explores the potential of exogenous auxins in the development of adventitious shoots and roots from shoot cuttings of* Couroupita guianensis *(Nagalingam), a threatened tree. Experiments were conducted to assess the effect of various concentrations of auxins on shoot and root morphological traits of stem cuttings in the greenhouse. Amongst the auxins tested, significant effects on number of shoot buds' induction and their growth were observed with *α*-Naphthalene Acetic Acid (NAA) treated nodal cuttings. Cent percentage of the stem cuttings of* C. guianensis* were rooted and shoots were induced when pretreated with 400 mg L^−1^ NAA for 5 min. Maximum 79% of stem cuttings responded to pretreatment of 300 mg L^−1^indole-3-butyric acid (IBA) for 5 min, and 75% of stem cuttings induced shoots with 400 mg L^−1^indole-3-acetic acid (IAA). Presence of at least 5 nodes on stem cuttings was found to be prerequisite for root and shoot induction. About 92% of plants were survived under natural soil conditions raised from the stem cuttings. This is the first report of vegetative propagation of* C. guianensis *through stem cuttings which could be used for conservation strategy and sustainable utilization of this threatened medicinal tree.

## 1. Introduction

*Couroupita guianensis* Aubl. (family Lecythidaceae) is commonly known as Cannonball tree, Nagalingam, Ayahuma, Kailaspati, Calabasse Colin, Bala de canon, and Carrion tree [1]. Due to its high medicinal value and absence of propagation system, the tree has been exploited indiscriminately, therefore facing a high risk of extinction, and was listed as threatened species worldwide in the IUCN red list [2, 3]. This large deciduous as well as evergreen tree grows to the height of 20 meters with simple clustered leaves at the juvenile part of the stem. The tree bears large cluster of cauliflorous racemose inflorescence with stunning fragrance. It produces amphisarcum, woody, indehiscent fruits with many seeds [4, 5].

This plant gathers medicinal importance due to the presence of eugenol, fernesol, nerol, tryptanthrine, indigo, indirubin, isatin, linoleic acid, *α*- and *β*-amirins, carotenoids, sterols, phenolic compounds, rutin, quercetin, kaempherol, famaricetin, luteolin, saponin, alkaloids, terpenoids, tannins, triterpenoids, anthocyanin, flavonoids, steroids, anthraquinones, citric acid, tartaric acid, couroupitine, and pelargonidin glycosides [6–8].

Traditionally the whole plant is used in various healing protocols of hemorrhage, piles, scabies, dysentery, scorpion sting, hypertension, tumors, malaria, odontalgia, inflammatory processes, kidney and stomach problems, allergies, ulcers, toothache, and skin diseases [9, 10]. The species has been explored for various biological life supporting properties like, antibiotic, antifertility, wound healing [11], immunomodulatory [12], anthelmintic [13], antinociceptive, antitumor [14], antipyretic, larvicidal, insecticidal, pesticidal [15], antiulcer, antiarthritic, antidiarrheal [16], antidiabetic [8], neuropharmacological, antioxidant, and anticancer activities [17].

The extraordinary medicinal properties coupled with poor reproduction ability and environmental disasters increased anthropogenic and livestock disturbances; the population of this species has been hampered in the study area [18]. The Government of Puducherry (India) has declared* C. guianensis *flower (Nagalingam flower) as the Official State Flower to conserve this valuable tree under natural habitats in the south India [19]. It has also been listed as rare flower and tree of India [5].

The conventional propagation of* C. guianensis *through seeds was hindered due to poor viability and germination frequency and recalcitrant nature of seeds [20, 21]. Therefore, it is necessary to develop alternative methods of propagation for conservation of this tree species.

Vegetative propagation via stem cuttings offers production of true-to-type plants in a short period of time and availability of superior individuals for large scale commercial plantation with quick productive gains [22, 23]. The exogenous use of auxins on stem cuttings for vegetative propagation has been successfully developed in much conservation prioritized (rare, endangered, and threatened) and commercially valuable plants such as* Pongamia pinnata* [22],* Ginkgo biloba* [23],* Hildegardia populifolia* [24],* Panax pseudoginseng* [25], and* Dillenia suffruticosa* [26].

The species can moreover be raised by in vitro propagation methods but it is very difficult to develop efficient micropropagation protocol for tree species due to the recalcitrant nature of the explants [27]. Phenolic compounds leached out in the nutritional medium are another hindrance in the development of in vitro methods for woody tree species because these compounds block the absorption of nutrients by the tissues. With this background, the present study aimed at the development of farmer/gardener friendly propagation protocol to ensure the population survival of the threatened species* C. guianensis*.

## 2. Materials and Methods

### 2.1. Plant Material and Preparation of Cuttings

Field surveys were conducted for selection of mature and superior plants of* C. guianensis* throughout the Coromandel coast of south India. The plants were identified with the help of “An excursion flora of Central Tamil Nadu and Carnatic” [28]. The stem cuttings of* C. guianensis* were harvested from the garden maintained healthy trees in the campus of the institute (Puducherry). The multinodal cuttings were harvested regularly with the help of sterilized wood cutter. Average length of the cuttings used for the study was 30–35 cm with multiple (7-8) nodes. All the leaves were removed from stem cuttings, and the cuttings were dipped in 0.1% bavistin (fungicide, BASF Ltd, Mumbai) solution (w/v) for 5 min subsequently washed with distilled water, and treated with root promoting auxins.

### 2.2. Preparation of Planting Medium (Soil Mixture)

Soil mixture plays important role in induction of roots from the cuttings. The planting medium is composed of a mixture of garden soil, red soil, vermicompost, and farm yard manure in equal ratio (1 : 1 : 1 : 1). All the four components of soil mixture were mixed properly to distribute nutrients uniformly to the growing cuttings and finally filled in the earthen pots.

### 2.3. Auxins Used in Roots and Shoots Induction

**Figure 1:**
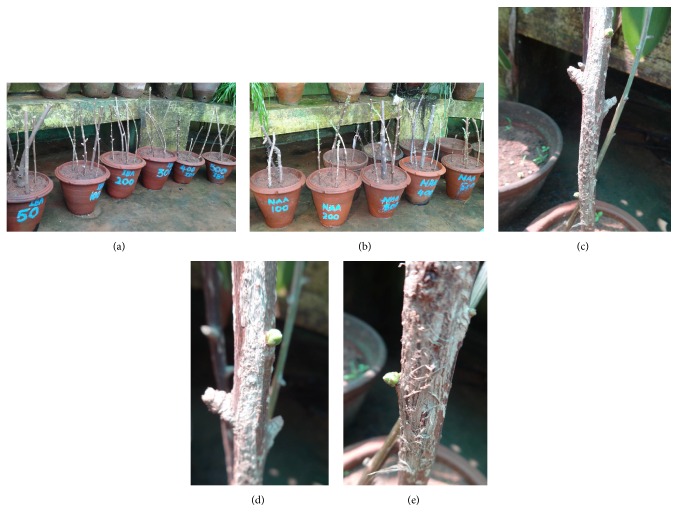
(a) Stem cuttings pretreated with different concentrations of IBA. (b) Stem cuttings pretreated with different concentrations of NAA. (c) IBA pretreated cuttings after 4 weeks. (d) IAA pretreated cuttings after 4 weeks. (e) NAA pretreated cuttings after 4 weeks.

To induce roots and shoots, the basal end (3.0 cm) of the cuttings was immersed in solutions of different concentrations (50, 100, 200, 300, 400, and 500 mg L^−1^) of auxins, that is, IAA, IBA, and NAA, for 5 min. The pulse treated cuttings were transferred to earthen pots containing soil mixture (Figures 1(a) and 1(b)). Homogenous nodal stem cuttings of comparable size were used for all the experiments.

### 2.4. Conditions of Adventitious Shoot Induction

Greenhouse plant growth unit lined with netted polysheets was used for adventitious shoot induction in all the stem cuttings selected in this study. The cuttings were maintained in the greenhouse at 28–30°C temperature and approximately 80–90% relative humidity. The cuttings were immersed for the same time in distilled water as control experiments.

### 2.5. Culture Methods

First irrigation to the pots containing stem cuttings was flooded and, thereafter, the cuttings were watered with fine jet sprayer once every day, until completion of the experiment. Cuttings were defined as “dead” when severely rotted and accompanied by discoloring, drooping, or bleaching. The propagation period for the experiment was 60 days.

### 2.6. Statistical Analysis

The experiments were conducted in randomized block design method with three replicates, each comprised of 5 cuttings. The phenotypic observations were taken periodically according to the time period of shoot induction for all the three treatments in greenhouse and in soil (control). The cuttings having at least one shoot were considered for various parameters such as rooting and shooting percentage and number and length of shoots. The data for shoots length were observed after 30 and 60 days of insertion of stem cuttings in the soil mixture. The data were analyzed statistically using SPSS v.16 (SPSS, Chicago, USA). The significance of differences among mean values was carried out using Duncan's multiple range test or paired sample* T* test at *P* < 0.05. The results are expressed as mean ± SE of three experiments.

## 3. Results and Discussion

The macropropagation techniques play an integral part of tree improvement programs and have been explored for the propagation of many economically valuable species particularly the rare, endangered, and threatened plant species [25]. The propagation system using stem cuttings can be a key step in vegetative propagation and minimizing the risk of declining the species. This is the first report which deals with the methods developed for proliferation of shoots and roots from stem cuttings of cannonball tree using auxins.

### 3.1. Regeneration Potential of Stem Cuttings

The results on response of shoot bud induction attributed to auxins were recorded after 60 days of planting in the soil mixture. The stem cuttings without any treatment (control) were failed to induce shoots and roots. The effect of exogenous auxins treatments on shoot bud induction efficacy of stem cuttings of* C. guianensis* is presented in Table 1. All cuttings were checked periodically for emergence of shoots or shoot primordial. Though the shoot buds were induced on all the treatments except control, the percentage of response varies with the type and concentration of auxin used. The differential morphophysiological response of stem cuttings on various auxins has been ascribed to the chemical nature of auxins and the mode of treatments [29–31].

**Figure 2:**
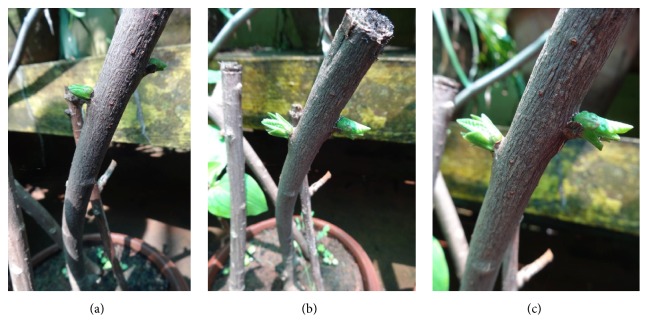
(a)–(c) NAA pretreated cuttings after 6 weeks.

It was observed that almost all the treatments except control were able to induce shoot buds and roots in stem cuttings, and the application of NAA was found to be effective than IBA and IAA in this study (Figures 1(c)–1(e)). Maximum shoot bud induction and rooting response were observed on woody cuttings pretreated with NAA at 400 mg L^−1^ concentration followed by 300 mg L^−1^ concentration of IBA. Shoot bud initiation in majority of cuttings was noticed after 6 weeks on all the auxins tested with tremendous differences in number and percentage response between the concentrations. The lowest concentration of NAA (50 mg L^−1^) was found to be less effective than the higher concentration (400 mg L^−1^) (Figures 2(a)–2(c)). The results are contrast with the reports on* Cedrus deodara* [32] and* Gingko biloba* [23], which revealed that the IBA was more effective for root induction in stem cuttings.

In case of IBA, lower concentration of auxins induced shoot buds from the nodes and gradual increase in number of shoot buds observed till 300 mg L^−1^; however, it was slightly less effective than the NAA. The exogenous application of IBA has been reported to enhance the speed of translocation and movement of sugar to the stem cuttings and promote root growth [33]. The response of cuttings on IAA was very poor with the values for average number of shoot buds, roots, and their length in comparison to other auxins (Table 2).

**Figure 3:**
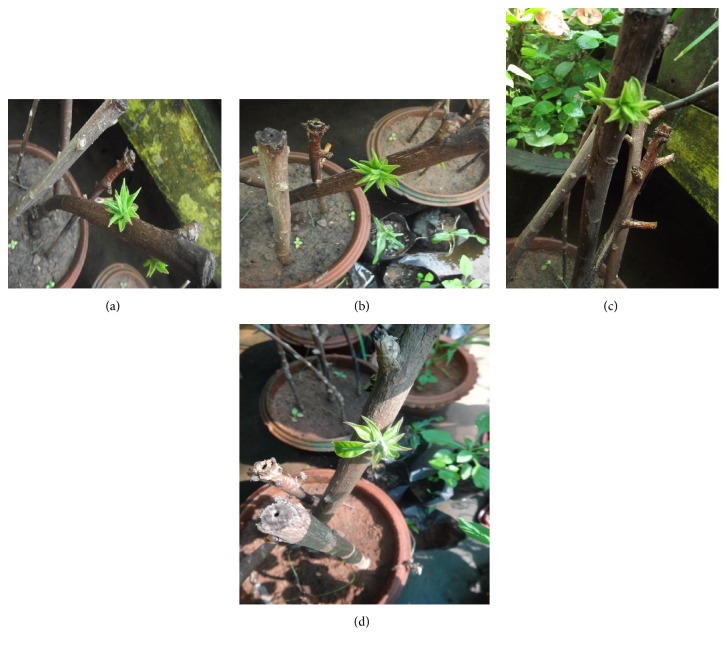
(a), (b), and (d) Different stages in NAA pretreated stem cuttings. (c) IBA treated stem cuttings after 8 weeks.

The stem cuttings maintained with the concentration of 400 mg L^−1^ NAA responded with 4.8 ± 0.17 shoot buds with the average length of 3.5 ± 0.12 cm (Figures 3(a), 3(b), and 3(d)). Maximum 98% of rooting with 3.6 ± 0.10 roots (3.0 ± 0.27 cm length) per cuttings was achieved at this concentration. There was a gradual increase in response of shoots and roots from 50 mg L^−1^ to 400 mg L^−1^ (Tables 1 and 2). The concentrations of IBA from 50 mg L^−1^ to 300 mg L^−1^ showed a significant increase in the number of shoots from 1.4 ± 0.29 to 3.6 ± 0.31 and average shoot length (from 0.5 ± 0.11 to 2.0 ± 0.25 cm) (Figure 3(c)). Similar results were observed in rooting experiments also. However, further increase in the concentration of NAA and IBA showed a significant reduction in the number of shoots and roots and average length of shoots and roots. The results obtained with IAA treatments were not impressive in terms of root and shoot induction than NAA and IBA. This reveals that the application of IAA exhibits poor root and shooting ability and is unable to respond well. Poor development of roots inhibits the success of vegetative propagation via cuttings [34]. Hartmann et al. [35] mentioned that the higher concentration of auxins inhibits the induction and elongation of roots and stimulates plant cell to produce ethylene.

The emergence of shoot buds from the stem cuttings of* C. guianensis* characterizes developmental plasticity by auxins and allows response to external environmental conditions. Auxins have been reported as central mediator of organ developments by promoting cell division, cell elongation, and cell differentiation [36]. The plantlets maintained under greenhouse during the acclimation process avoid losses and ensure plant uniformity [37]. The stem cuttings have been slowly phased into greenhouse conditions due to high relative humidity, less temperature, and irradiance.

The stem cuttings with shoots were carefully taken out from the pots and transferred to the field under natural conditions. The further growth rate was measured in terms of increase in number of leaves/node, shoot length, and percent survival of cutting-raised plants in the field environment. About 92% rooted stem cuttings were successfully survived under natural field conditions.

## 4. Conclusion

A protocol for vegetative propagation of* C. guianensis* was optimized with subsequent development of plants using stem cuttings as source material. The stem cuttings pretreated with 400 mg L^−1^ NAA for 5 min showed 100% response on planting medium containing garden soil, red soil, vermicompost, and farm yard manure after 60 days of planting in greenhouse conditions. The optimized protocol can be used to develop healthy and profuse root system with shoot bud development and further proliferation. This method can play key role in rapid supply of quality planting material of the Cannonball plants and help in minimizing the current challenge of its conservation.

## Figures and Tables

**Table 1:** The effect of auxins on shoot bud induction in stem cuttings of *C. guianensis* after 60 days of treatment under greenhouse condition.

Concentration of auxins (mg L^−1^)			Shoot bud induction response (%)	Average number of shoots per cutting (mean ± SE)	Average length of shoots (mean ± SE)
IAA	IBA	NAA			
50	—	—	33	1.0 ± 0.11^a^	1.1 ± 0.17^b^
100	—	—	48	1.9 ± 0.18^ab^	1.4 ± 0.00^b^
200	—	—	56	2.3 ± 0.20^b^	1.5 ± 0.28^c^
300	—	—	62	2.8 ± 0.12^c^	2.2 ± 0.10^d^
400	—	—	75	2.5 ± 0.00^bc^	1.8 ± 0.21^c^
500	—	—	70	2.0 ± 0.14^b^	1.4 ± 0.19^b^
—	50	—	59	1.4 ± 0.29^a^	0.5 ± 0.11^a^
—	100	—	64	2.2 ± 0.31^b^	1.0 ± 0.14^b^
—	200	—	72	3.0 ± 0.14^d^	1.4 ± 0.00^b^
—	300	—	79	3.6 ± 0.31^d^	2.0 ± 0.25^d^
—	400	—	70	3.1 ± 0.14^d^	1.8 ± 0.10^c^
—	500	—	67	2.6 ± 0.27^c^	1.1 ± 0.15^b^
—		50	66	2.7 ± 0.12^c^	0.9 ± 0.10^ab^
—		100	71	3.0 ± 0.20^d^	1.2 ± 0.18^b^
—		200	87	3.4 ± 0.16^d^	2.0 ± 0.14^d^
—		300	92	4.0 ± 0.00^e^	2.8 ± 0.29^e^
—		400	100	4.8 ± 0.17^f^	3.5 ± 0.12 ^g^
—		500	96	3.5 ± 0.24^d^	3.2 ± 0.00^f^

Note: stem cuttings with at least one shoot bud were considered for calculating percentage of shoot bud induction; SE: standard error; level of significance *P* < 0.05. All values are an average of 10 replicates. The mean values represented in corresponding column followed by the same alphabets are not significantly different. Here “a” represents/considered as the lowest value and “g” as highest value.

**Table 2:** The effect of auxins on rooting of *C. guianensis* stem cuttings.

Concentration of auxins (mg L^−1^)			Rooting response (%)	Average number of roots per cutting (mean ± SE)	Average length of roots (mean ± SE)
IAA	IBA	NAA			
50	—	—	42	1.6 ± 0.16^a^	1.0 ± 0.23^a^
100	—	—	49	2.1 ± 0.22^b^	1.3 ± 0.20^a^
200	—	—	54	2.5 ± 0.19^b^	1.7 ± 0.12^b^
300	—	—	69	2.9 ± 0.11^c^	2.0 ± 0.16^c^
400	—	—	79	3.0 ± 0.29^c^	1.9 ± 0.20^b^
500	—	—	72	2.6 ± 0.15^b^	1.6 ± 0.11^b^
—	50	—	64	1.9 ± 0.20^a^	1.0 ± 0.27^a^
—	100	—	67	2.3 ± 0.28^b^	1.3 ± 0.10^a^
—	200	—	76	2.8 ± 0.23^c^	1.7 ± 0.12^b^
—	300	—	81	3.3 ± 0.11^d^	2.3 ± 0.20^d^
—	400	—	77	3.0 ± 0.10^c^	1.5 ± 0.15^ab^
—	500	—	60	2.5 ± 0.15^b^	1.0 ± 0.29^a^
—		50	73	2.2 ± 0.19^b^	1.9 ± 0.31^c^
—		100	86	2.7 ± 0.27^b^	2.0 ± 0.20^c^
—		200	91	3.0 ± 0.00^c^	2.7 ± 0.13^d^
—		300	93	3.2 ± 0.10^d^	2.7 ± 0.11^d^
—		400	98	3.6 ± 0.10^e^	3.0 ± 0.27^e^
—		500	91	3.1 ± 0.00^c^	2.4 ± 0.00^c^

Note: stem cuttings with at least one root were considered for calculating percentage of rooting; SE: standard error; level of significance *P* < 0.05. All values are an average of 10 replicates. The mean values represented in corresponding column followed by same alphabets are not significantly different. Here “a” represents/considered as the lowest value and “g” as highest value.

## Abbreviations

IAA: Indole-3-acetic acid
IBA: Indole-3-butyric acid
NAA: *α*-Naphthalene Acetic Acid.
